# Using biologically based objectives to optimize boost intensity‐modulated radiation therapy planning for brainstem tumors in dogs

**DOI:** 10.1111/vru.12815

**Published:** 2019-10-10

**Authors:** Valeria Meier, Jürgen Besserer, Carla Rohrer Bley

**Affiliations:** ^1^ Division of Radiation Oncology, Small Animal Department, Vetsuisse Faculty University of Zurich Zurich Switzerland; ^2^ Department of Physics University of Zurich Zurich Switzerland; ^3^ Radiation Oncology Hirslanden Clinic Zurich Switzerland

**Keywords:** biologically based treatment planning, brain, gEUD, IMRT, SIB

## Abstract

Irradiated brain tumors commonly progress at the primary site, generating interest in focal dose escalation. The aim of this retrospective observational study was to use biological optimization objectives for a modeling exercise with simultaneously‐integrated boost IMRT (SIB‐IMRT) to generate a dose‐escalated protocol with acceptable late radiation toxicity risk estimate and improve tumor control for brainstem tumors in dogs safely. We re‐planned 20 dog brainstem tumor datasets with SIB‐IMRT, prescribing 20 × 2.81 Gy to the gross tumor volume (GTV) and 20 × 2.5 Gy to the planning target volume. During the optimization process, we used biologically equivalent generalized equivalent uniform doses (gEUD) as planning aids. These were derived from human data, calculated to adhere to normal tissue complication probability (NTCP) ≤5%, and converted to the herein used fractionation schedule. We extracted the absolute organ at risk dose‐volume histograms to calculate NTCP of each individual plan. For planning optimization, gEUD_(a = 4) _= 39.8 Gy for brain and gEUD_(a = 6.3) _= 43.8 Gy for brainstem were applied. Mean brain NTCP was low with 0.43% (SD ±0.49%, range 0.01‐2.04%); mean brainstem NTCP was higher with 7.18% (SD ±4.29%, range 2.87‐20.72%). Nevertheless, NTCP of < 10% in brainstem was achievable in 80% (16/20) of dogs. Spearman's correlation between relative GTV and NTCP was high (ρ = 0.798, *P* < .001), emphasizing increased risk with relative size even with subvolume‐boost. Including biologically based gEUD values into optimization allowed estimating NTCP during the planning process. In conclusion, gEUD‐based SIB‐IMRT planning resulted in dose‐escalated treatment plans with acceptable risk estimate of NTCP < 10% in the majority of dogs with brainstem tumors. Risk was correlated with relative tumor size.

**Abbreviations**
CI: conformity index
CTV: clinical target volume
*D*_2_: near‐maximum dose
*D*_50_: median dose
*D*_98_: near‐minimum dose
EUD: equivalent uniform dose
gEUD: generalized equivalent uniform dose
GI: gradient index
GTV: gross tumor volume
ICV: intracranial volume
IG‐IMRT: image‐guided IMRT
NTCP: normal tissue complication probability
OAR: organ at risk
PTV: planning target volume
RT: radiation therapy
SIB: simultaneously integrated boost

## 1 INTRODUCTION

Irradiated brain tumors in dogs commonly relapse at the site of the primary tumor, generating an interest to escalate radiation dose in order to increase tumor control.^1^, ^2^, ^3^, ^4^ A careful approach to increase dose can be achieved with simultaneously integrated boost (SIB) radiation therapy (RT), where a subvolume such as the macroscopic/gross tumor volume (GTV) receives an additional, higher boost dose. The prescribed dose to the clinical (CTV) and planning target volume (PTV) remains the same as the previously used protocol (ie, in the regular range) and the dose to the surrounding normal tissue – the tissue at risk for side effects – should therefore not be markedly higher. This concept of dose escalation to a subvolume has been found to increase local tumor control in human studies, while keeping risk for normal tissue toxicity in the immediate surrounding tissue comparable.^3^, ^5^

The region of the brainstem is a sensitive area in terms of radiation tolerance and therefore a critical location to increase dose.^6^, ^7^, ^8^, ^9^ In a previous study, we computed the theoretical risk for late toxicity for intracranial tumors in the dog treated with a commonly used protocol of 20 × 2.5 gray (Gy) and compared this to the risk of a new protocol with fewer, larger fractions.^9^ The original 20‐fraction protocol bore a low risk of potentially fatal complications (brain/brainstem necrosis), with normal tissue complication probability (NTCP) of ≤5% in most of dogs with brainstem tumors. This low complication risk mirrors the clinical experience in brain and brainstem tumors treated with finely fractionated RT.^1^, ^2^, ^4^, ^9^ The technical advancement of image‐guided IMRT (IG‐IMRT) and its resulting high precision encourages delivering a higher total radiation dose. Higher doses can be given under the premises that normal tissue constraints are met. These constraints are used for prioritizing the dose‐distribution in tumor‐surrounding organs at risk in IMRT treatment optimization. Recently, different vendors introduced models with biologically based planning constraints for human patients into treatment planning software. As an advantage, biologically based parameters consider tissue‐specific characteristics – parallel, serial, or parallel‐serial architecture of organs at risk – into the planning process.^10^ Hence, compared to the simple physical dose or dose‐volume constraint‐based optimization, biologically based constraints account for the sensitivity of organs at risk volumetrically.

With the aim to improve tumor control for brainstem tumors in dogs safely, we used biological optimization objectives for theoretical treatment planning in a SIB‐IMRT protocol. We re‐planned dog datasets with a 20 × 2.5 Gy radiation protocol with a subvolume boost to the GTV of 20 × 2.81 Gy. As biological optimization objective, we used generalized equivalent uniform doses (gEUD) as treatment planning aids and subsequently performed NTCP computations to estimate the risk of late toxicity for each individual plan. Our objective was to generate a dose‐escalated protocol with an acceptable late radiation toxicity risk estimate.

## 2 METHODS

### 2.1 Dog and tumor characteristics

The study was a retrospective, observational design. Computed tomography datasets of client‐owned dogs with brainstem tumors formerly treated with radiation therapy at the authors' institution between 2012 and 2018 were included in the study. Presumptive diagnosis of a brainstem tumor had been made by diagnostic imaging (either with MRI and CT or with CT alone). Datasets were included into the study, if a brainstem tumor (defined as a tumor between the *dorsum sellae* and petrosal crests and the *foramen magnum)* was present. Datasets with tumors in the cerebellum, not adjacent to the brainstem, were excluded by an ACVR(RO)‐certified veterinary radiation oncologist (VM). Additional dog characteristics such as breed, sex, weight, age, presumed tumor type, tumor, and organs at risk volume were retrieved from the medical records.

### 2.2 Technical equipment

As part of the inclusion criteria, all datasets consisted of two CT studies with 2 mm contiguous slices: a native 3D CT dataset (Brilliance CT 16‐slice, Philips Health Care Ltd, Best, the Netherlands), followed by a co‐registered intravenous contrast study (Accupaque 350, 755 mg Iohexol, 350 mg/I/mL, osmolality of 780 mOsmol/kg, GE Healthcare AG, Glattbrugg, Switzerland) as previously described.^11^ Co‐registration with appropriate MRI images (contrast‐enhanced T1 sequences) was performed if available in order to facilitate contouring.

For this planning study, the planning target volume, planning and treatment technique, and equipment were chosen to reflect how radiation therapy is delivered at the authors' institution, with a 6 MV linear accelerator (Clinac iX, Varian, Palo Alto, California, USA), using a 120‐leaf dynamic multileaf collimator (Millenium MLC™), assuming high accuracy and precision in target localization is achieved due to rigid patient positioning with an individually shaped vacuum cushion immobilizing the thorax and front limbs and a bite block and assuming daily image‐guidance is used for setup verification, with daily kilovolt (kV) orthogonal radiographs and twice a week kV‐CBCT. For treatment planning, the External Beam Planning system (Eclipse™ Planning system, version 15.1; Varian Oncology Systems, Palo Alto, California, USA) with the Photon Optimizer (version 15.1), Anisotropic Analytical Algorithm (version 15.1), and heterogeneity correction was used.

### 2.3 Contouring

Target volumes were delineated as follows: the original GTV included the visible tumor as contrast‐enhancing area seen on co‐registered contrast‐enhanced CT or MRI images; the CTV accounting for microscopic disease included a 2 mm isotropic expansion from GTV into intracranial soft tissue; for the PTV, a 2 mm isotropic expansion from CTV was performed. The organs at risk (OAR) brain and brainstem were delineated as previously described^9^, ^12^: (a) intracranial volume (ICV; until the caudal end of the *foramen magnum*), (b) brain volume (equal to the ICV minus the brainstem and gross tumor volume), (c) brainstem volume (equal to the ICV minus the brain and gross tumor volume). In order to assess relative target volume size, we calculated the relative target volumes, that is, ratios of the target volume to the organ at risk (eg, GTV/brainstem).

### 2.4 New SIB protocol

The boost dose to the GTV was computed as follows: for this modeling exercise, we wanted to administer a higher total dose, but limit it to a value that would not exceed the tolerance of the OAR brain (as most brain tumors in dogs are in the brain and fewer in the brainstem area). According to the summary of Emami from 2013, the tolerance of brain (with a < 5% rate of symptomatic necrosis) is 65 Gy.^13^ Divided by the commonly used 30 fractions for human brain tumor patients, this equals to a dose per fraction of 2.17 Gy. This was recalculated into the equivalent dose in 2 Gy fractions (EQD2) with an alpha/beta value of 2 Gy (for late toxicity), yielding a total dose of 68 Gy. In keeping this value constant, the new protocol with 20 instead of 30 fractions was 20 × 2.81 Gy (EQD2 = 68 Gy).

### 2.5 Treatment planning

An ACVR(RO) board‐certified veterinary radiation oncologist (CRB or VM) re‐planned all dog datasets. Inverse planning was carried out using seven coplanar fields. The SIB‐IMRT protocol prescribed 20 × 2.5 Gy (total physical dose 50 Gy) to the PTV and a SIB of 20 × 2.81 Gy (total physical dose 56.2 Gy (+12.4%)) to the GTV. We aimed for a rapid dose‐fall off between the outer edge of the GTV and the PTV using planning helper structures. Those helper structures are used to achieve optimization objectives and consisted of a ring structure (margin of 1 cm surrounding the PTV cropped 2‐3 mm from the outside of the PTV), a BodyminPTV structure (body structure cropped 2‐3 mm from the outside of the PTV), a dose fall‐off structure (PTV cropped 2–3 m from the outside of the GTV), or an OARminPTV structure (brain or brainstem cropped 2 mm from the outside of the PTV) according to the radiation oncologist's preference. For IMRT prescribing and reporting, we followed the dose‐volume specifications as recommended by the ICRU report 83.^14^, ^15^ For the SIB, we set requirements for the GTV as follows: *D_98%_* (*D_near‐min_)* to *≥*53.4 Gy and *D_2%_* (*D_near‐max_)* to ≤59.0 Gy. This *D_near‐min_* corresponds to the minimal dose coverage of D_98_ with 95% of total prescribed dose. For the PTV, we set *D_98%_* (*D_near‐min_)* to ≥47.5 Gy and *D_2%_* (*D_near‐max_)* to the high ≤59.0 Gy, as it included the GTV dose. Dose was prescribed to the median (D_50%_), as suggested by ICRU report 83.^15^, ^16^, ^17^

### 2.6 Treatment plan assessment

For assessment of dose homogeneity, we calculated a homogeneity index of the GTV (HI_GTV_) as follows^15^, ^16^, ^17^:

$$\mathrm{Homogeneity}\;\mathrm{Index}\;\left(\mathrm{HI}\right)\;=\;\frac{\left(D_{2}-D_{98}\right)}{D_{50}}$$

where *D_2_* is the near‐maximum dose, *D*_98_ is the near‐minimum dose, and *D*_50_ the median dose. A homogeneity index of 0 indicates an almost homogeneous absorbed‐dose distribution.

In order to characterize how well the high‐dose area of the dose is shaped around the PTV, we computed a conformity index (CI) for the PTV (CI_PTV_) with the following formula^12^, ^18^, ^19^:

$$\mathrm{Conformity}\;\mathrm{Index}\;\left(\mathrm{CI}\right)\;=\;\frac{T{V_{\mathrm{PIV}}}^{2}}{\left(\mathrm{TV}\times \mathrm{PIV}\right)}$$

Where TV_PIV_ is the target volume (PTV) covered with the prescription dose (50 Gy), TV is the target volume (PTV), and the PIV is the prescription isodose volume (50 Gy). A CI of 1 indicates that there is a high degree of conformity.

To assess the steepness of dose fall‐off outside of the PTV, we calculated a gradient index (GI) for the PTV (GI_PTV_) as follows^12^, ^20^:

$$\mathrm{Gradient}\;\mathrm{Index}\;\left(\mathrm{GI}\right)\;=\;\frac{V_{50}}{V_{100}}$$

where V_50_ is the volume receiving 50% of the PTV prescription dose (25 Gy) divided by the volume receiving 100% of the prescription dose (50 Gy). A low GI indicates a steep dose gradient (rapid dose fall‐off).

The indices were used to compare plans after calculation.

### 2.7 Biologic modeling and toxicity estimate

The concept of equivalent uniform dose (EUD) assumes that two different radiation dose distributions are equivalent, if they produce the same radiobiological effect. According to this concept it is “(…) assumed that a notionally uniform dose, administered to the tumor, produces the same radiobiological effect as the non‐uniform dose distribution of interest.”^21^

Niemierko (1997/1999) proposed the phenomenological formula for the generalized EUD (gEUD) based on the power law dependence of the response of biological systems to a stimulus^21^, ^22^:

$$\mathrm{Generalized}\;\mathrm{equivalent}\;\mathrm{uniform}\;\mathrm{dose}\;\left(\mathrm{gEUD}\right)\;={\left(\sum_{i}v_{i}D_{i}^{a}\right)}^{1/a}\;$$

With this formula the, gEUD can be calculated from the dose volume pairs $\{v_{i},D_{i}$} of the differential dose‐volume histograms, where $v_{i}$ is the partial volume irradiated to dose $\;D_{i}$ in bin number *i*. The parameter *a* is particular to each organ and describes the volumetric dependence of the dose‐response relationship that has so far only been derived in human patients. We took parameter sets from Burman et al. (a = 6.25, m = 0.14, TD_50 _= 65 for brainstem, and a = 4, m = 0.15, TD_50 _= 60 for brain, alpha/beta value = 2), which are based on fits to human normal tissue data compiled by Emami et al. as previously described. We plotted gEUD against NTCP from a 30 × 2 Gy protocol used for human glioma irradiation.^8^, ^9^, ^17^, ^23^, ^24^ In order to adjust for fraction size and fraction number in the new SIB protocol, the parameter gEUD was converted to a biologically equivalent gEUD using the linear‐quadratic model. The biologically equivalent gEUD for NTCP ≤5% for brainstem and brain was then used as an estimate for an upper limit (upper gEUD) during plan optimization.

We then extracted dose‐volume data of organs at risk for each individual plan and used them for NTCP computations with the Lyman equivalent model and gEUD for brainstem and brain as prior described in detail.^9^, ^24^, ^25^ NTCP was correlated to the different ICVs (GTV/brainstem, GTV/ICV) as previously reported.^9^

### 2.8 Statistical analysis

Statistical tests were selected and performed by an observer with statistical expertise. Data were coded in a spreadsheet (Excel, version 14.7.7, Microsoft Corp., Redmond, WA) and analyzed with a commercial statistical software package (IBM^®^ SPSS^®^ Statistics, Version 24, IBM Corp., Armonk, New York). Descriptive statistics such as absolute and relative frequencies as well as mean (median) and SD (interquartile range (IQR)) were computed. Spearman's correlation was used to test for associations between target and brain volumes and NTCP. Results of statistical analysis with *P*‐values *<* .05 were interpreted as statistically significant.

## 3 RESULTS

CT datasets of 20 dogs were included in the sample and used for treatment planning. Demographics of all dogs are depicted in Table 1. All except two dogs had an imaging diagnosis of meningioma (one was confirmed at necropsy); in two dogs, a schwannoma/neurofibroma originating from the trigeminal nerve but with marked intracranial component was suspected.

**Table 1:** Dog demographics

Breed	Pure breed: n = 11
	Mixed breed: n = 9
Sex	Female: n = 2
	Female spayed: n = 7
	Male: n = 3
	Male neutered: n = 8
Weight	Mean: 24.1 kg (SD ±8.7; 95%CI, 20.3‐27.9)
	Median: 25.3 kg (IQR: 13.1)
Age	Mean: 9.6 years (SD ±3.0; 95%CI, 8.1‐11.0)
	Median: 9.4 years (IQR: 5.0)
Presumed tumor type (based on MRI diagnosis)	Meningioma: n = 18
	Schwannoma/neurofibroma: n = 2

Abbreviations: 95%CI, confidence interval; MRI, magnetic resonance imaging.

The mean target volumes were as follows: GTV was 2.3 cm^3^ (SD ±1.2), CTV 4.6 cm^3^ (SD ±2.2), and PTV 9.0 cm^3^ (SD ±3.8). The mean ratio of the GTV to the brainstem (relative tumor volume, GTV_brainstem_) was 0.33 (SD ±0.17). All absolute and relative target volumes and brainstem and brain (OAR) volumes are depicted in Table  2A and 2B.

**Table 2A:** Organ at risk volume, absolute target volumes of all CT datasets

Absolute volume mean (±SD; 95% CI) median (IQR) (cm^3^)					
Target volume			OAR		
GTV	CTV	PTV	Brainstem	Brain	ICV
2.3 (±1.2; 1.8, 2.9)	4.6 (±2.2; 3.5, 5.6)	9.0 (±3.8; 7.2, 10.8)	7.1 (±1.6; 6.4, 7.8)	80.6 (±12.4; 74.8, 86.4)	90.0 (±13.3; 83.8, 96.3)
2.2 (1.4)	4.3 (2.7)	9.3 (5.2)	7.1 (1.6)	78.1 (20.9)	88.0 (21.6)

Abbreviations: 95%CI, 95% confidence interval; CTV, clinical target volume; GTV, gross tumor volume; ICV, intracranial volume; OAR, organ at risk; PTV, planning target volume.

**Table 2B:** Organ at risk volume, relative target volumes of all CT datasets

Relative volume mean (±SD; 95% CI) median (IQR)				
Target volume/OAR				
Relative GTV_brainstem_	Relative CTV_brainstem_	Relative PTV_brainstem_	Relative GTV_brain_	Relative GTV_ICV_
(GTV/brainstem)	(CTV/brainstem)	(PTV/brainstem)	(GTV/brain)	(GTV/ICV)
0.3 (±0.2; 0.3, 0.4)	0.7 (±0.3; 0.5, 0.8)	1.3 (±0.6; 1.0, 1.6)	0.03 (±0.01; 0.02, 0.03)	0.03 (±0.01; 0.02, 0.03);
0.3 (0.3)	0.6 (0.5)	1.2 (0.9)	0.02 (0.02)	0.02 (0.02)

Abbreviations: 95%CI, 95% confidence interval; CTV, clinical target volume; GTV, gross tumor volume; ICV, intracranial volume; OAR, organ at risk; PTV, planning target volume.

For planning optimization, the gEUD_(a = 6.3) _= 43.8 Gy for brainstem and gEUD_(a = 4) _= 39.8 Gy for brain were derived (chosen to keep NTCP ≤5%) and used during plan optimization. Absorbed doses and indices are shown in Tables 3 and 4.

**Table 3:** Absorbed dose information of all CT datasets

Absorbed doses mean (±SD; 95% CI); median (IQR)^43^														
Brainstem			Brain			GTV			CTV			PTV		
*D*_50%_	*D*_98%_	*D*_2%_	*D*_50%_	*D*_98%_	*D*_2%_	*D*_50%_	*D*_98%_	*D*_2%_	*D*_50%_	*D*_98%_	*D*_2%_	*D*_50%_	*D*_98%_	*D*_2%_
31.10	5.56	53.71	6.79	0.31	51.34	56.20	54.15	57.58	55.27	51.28	57.49	53.81	48.21	57.26
(±11.73;	(±6.99;	(±0.81;	(±7.43;	(±0.27;	(±2.88;	(±0.02;	(±0.57;	(±0.77;	(±0.46;	(±0.87;	(±0.64;	(±0.77;	(±0.76;	(±0.56;
25.61,	2.29,	53.34,	3.31,	0.19,	49.99,	56.19,	53.88,	57.22,	55.05,	50.87,	57.18,	53.45,	47.86,	57.0,
36.59);	8.84);	54.09);	10.26);	0.44);	52.69);	56.2),	54.42);	57.94);	55.48);	51.68);	57.79);	54.17);	48.57);	57.52);
33.50	3.27	53.84	3.32	0.23	51.71	56.2	54.17	57.50	55.31	51.25	57.46	53.91	47.92	57.24
(11.75)	(4.40)	(0.90)	(8.50)	(0.15)	(2.33)	(0.03)	(1.0)	(0.89)	(0.76)	(1.38)	(0.48)	(1.27)	(0.94)	(0.60)

Abbreviations: 95%CI, 95% confidence interval; CTV, clinical target volume; D_50%_, median dose; D_98%_, near‐minimum dose; D_2%_, near‐maximum dose; GTV, gross tumor volume; PTV, planning target volume.

**Table 4:** Homogeneity, conformity and gradient indices of all CT datasets

Indices mean (±SD); median (95% CI; IQR)		
GTV	PTV	
HI_GTV_	CI_PTV_	GI_PTV_
0.06 (±0.02;	0.65 (±0.08;	7.2 (±1.45;
0.05, 0.07);	0.62, 0.69);	6.55, 7.91);
0.07 (0.03)	0.66 (0.11)	6.82 (2.29)

Abbreviations: CI, conformity index; 95%CI, 95% confidence interval; GI, gradient index; GTV, gross tumor volume; HI, homogeneity index; PTV, planning target volume.

Mean and median NTCP for brain were low, with 0.43% (SD ±0.49; 95% CI 0.20‐0.66) and 0.32% (IQR 0.36), respectively. Mean and median NTCP for brainstem were higher, with 7.18% (SD ±4.29, 95% CI, 5.17‐9.19) and 6.42% (IQR 5.79), respectively. NTCP for brain was below 2.04% in all dogs and only 3/20 dogs had NTCP of > 1% for brain. The aspired low NTCP of ≤5% for brainstem was achievable in 35% (7/20) dogs, a more reasonable NTCP of ≤10% in 80% (16/20) dogs. During the planning process, the gEUD value for brain could be met in all dogs. For brainstem, however, meeting the gEUD, we aimed at was only possible in 15 of 20 dogs. In seven of these 15 dogs, effective NTCP was low (≤5%), whereas in the remaining eight dogs, it was higher than 5%; this is shown in Table 5.

**Table 5:** The biologically based optimization objective gEUD and NTCP

Patient Number	NTCP_brainstem_ (%)	Effective gEUD_brainstem_ 43	gEUD_brainstem_ Could be met 1 = yes/0 = no	NTCP_brain_ (%)	Effective gEUD_brain_ 43	gEUD_brain_ Could be met 1 = yes/0 = no
1	20.72	47.89	0	1.10	32.77	1
2	3.13	40.01	1	0.12	27.21	1
3	3.63	40.53	1	0.00	19.77	1
4	8.41	43.66	1	0.48	30.51	1
5	5.84	42.23	1	0.62	31.16	1
6	7.61	43.26	1	0.46	30.42	1
7	3.40	40.33	1	0.33	29.58	1
8	9.90	45.35	0	0.02	23.20	1
9	6.10	42.40	1	0.13	27.34	1
10	8.50	43.71	1	0.36	29.77	1
11	5.12	41.75	1	2.04	34.58	1
12	6.74	42.78	1	0.30	29.33	1
13	10.19	44.47	0	1.09	32.72	1
14	10.42	44.57	0	0.35	29.68	1
15	12.75	45.52	0	0.48	30.53	1
16	2.90	39.78	1	0.03	24.27	1
17	4.15	40.99	1	0.15	27.67	1
18	2.87	39.71	1	0.26	28.99	1
19	4.22	41.06	1	0.09	26.48	1
20	6.96	42.91	1	0.13	27.34	1

Abbreviations: gEUD, generalized equivalent uniform dose; NTCP, normal tissue complication probability.

On Spearman's correlation, a high correlation between the relative GTV compared to the brainstem and NTCP of the brainstem (ρ = 0.798, *P* < .001) and between the relative GTV compared to the ICV and NTCP of the brainstem (ρ = 0.741, *P* < .001) was found. Hence, even though the boost dose is given to a subvolume only, there is a volume‐dependent increase in risk.

## 4 DISCUSSION

Radiation therapy leads to durable clinical and image‐based response in dogs with intracranial tumors.^1^, ^2^, ^4^, ^26^ In general, however, the tumors progress locally after a period of 1.5‐2 years.^1^, ^4^, ^26^ The time span to progression might increase with a higher dose of radiation.^3^ Currently, the total dose applied in dogs is relatively low, (most common range of EQD2 _alpha/beta 10_: 46.7‐52.1 Gy) and might be a reason for local progression or relapse.^1^, ^2^, ^4^, ^26^ In order to respect the sensitivity of normal brain and especially brainstem to high doses of radiation, we estimated the risk of toxicity, if only the macroscopic subvolume were to receive a higher boost dose. We used a boost total dose of 56.2 Gy in 20 fractions, which is 12.4% higher compared to the regular total dose of 50 Gy. If applying the EQD2 calculation for better comparison of regular compared to boost protocols, this adds up to a +15.2% boost (EQD2_50Gy; alpha/beta 10 _= 52.1 Gy and EQD2_56.2_ _Gy; alpha/beta 10 _= 60 Gy).^27^ As the slope of the sigmoidal dose‐response curve is steep at higher doses, such a dose difference can increase tumor control substantially.^3^

If applied with a subvolume boost, the planned dogs’ risk probability (NTCP) for brain injury was low with 0.43%. For brainstem, however, mean NTCP with 7.18% was slightly higher than the often‐used cut‐off of <5%. Depending on the volume of the tumor, the high boost dose exceeded brainstem tolerance and led to higher NTCP in some dogs. In one dog, the NTCP was even larger than 20% (patient 1 in Table 5). This dog had a broad‐based space‐occupying lesion located at the ventral aspect of the caudal fossa, extending through almost all its length, from the caudal aspect of the sella turcica to almost the level of the foramen magnum and causing marked mass effect to the brain stem. Of the included dogs, however, 35% would bear a low risk (NTCP < 5%) of late brainstem toxicity, while 80% of the dogs would bear a still “reasonable” risk estimate with NTCP < 10%. We calculated the boost dose using tolerance doses for human brain mentioned in Emami 2013.^13^ These tolerance parameter sets are mainly based on old data and might be overly conservative, that is, overestimating the risk. The true occurrence of late toxicity can therefore only be explored in a prospective clinical trial.

The risk correlated strongly with the relative size of the tumor (relative GTV). The tumor sizes comprised about one‐third of brainstem size (mean, median 2.2 cm^3^ (95% CI, 1.75‐2.86) and 2.3 cm^3^ (SD ±1.18), respectively) and were in a similar range as in previous studies (median 3.1 cm^3^, means of 2.8 to 3.2 cm^3^).^1^, ^4^, ^26^ The brains of dogs are small, with mean volumes of 81‐87 cm^3^.^1^, ^9^ Compared to human brains, this corresponds to a ratio of about 1:13. Radiation treatments, however, are delivered with the same accuracy in human and veterinary medicine given similar equipment and quality assurance. The high accuracy with IG‐IMRT allows for small PTV margins to account for inter‐ and intra‐fraction variability as position can be verified on a daily basis. This accuracy and the possibility for fast dose fall‐off in surrounding normal tissue are ideal for treating tumors in this location. As the target volumes (boost volume, CTV, and PTV) are often relatively small, however, it is possible that the desired fast dose fall‐off within such small margins cannot be achieved for technical/physical reasons. In consequence, if the dose from the boost to the regular dose applied in the PTV does not drop fast enough, the risk for toxicity to the surrounding tissue (organs at risk) will increase. The plans we generated for this study resulted in a homogenous dose distribution in the GTV, with median and mean homogeneity indices close to zero (0.06 and 0.07, respectively). The mean and median conformity indices were 0.65 and 0.66, respectively, a bit lower than in a previous study with stereotactic brain tumor treatment, but consistent with conformal treatment planning (according to van't Riet et al.: CI > 0.6).^12^, ^19^ However, the high mean and median gradient indices (6.55 and 6.82, respectively) indicate a rather slow dose fall‐off. This explains the volume dependence of the NTCP: a higher dose surrounding the PTV can occur (due to small margins between GTV and PTV), even if the boost dose is given to a subvolume only. Or this can in part be explained because we prioritized sparing of intracranial organs at risk and allowed a slower dose fall‐off in surrounding bone or muscle.

We added the biologically based optimization criterion of gEUD for the organs at risk (brain and brainstem) during the treatment planning process. However, the treatment plans – especially PTV – adhered also to the physical constraints of proper IMRT planning. We prioritized physical constraints in our planning and in five of 20 dogs, gEUD parameter for brainstem could not be met without compromising physical PTV constraints. Nevertheless, compared to plans optimized on physical constraints, adding biologically based objectives was found to produce plans with lower NTCP values for various types of cancer in humans.^28^, ^29^, ^30^ Overall, biologically based treatment planning based on the gEUD was reported to be superior in human prostate, head and neck, cervical carcinoma, and other tumor patients when compared to simple physical dose or dose‐volume criteria. As one of the features of gEUD, it impacts the whole DVH curve and not only point doses.^28^, ^29^, ^30^, ^31^, ^32^, ^33^, ^34^ If a low *a* value is used in the formula calculating gEUD, it will influence the mean dose (to protect a parallel organ, such as lung) and a high *a* value will influence the maximum dose level (used for protection of a serial organ, such as spinal cord). Caused by these parameters, gEUD respects the organization of functional subunits and architecture of the organ at risk and therefore serves as a single organ‐specific parameter for biological response. Contrary to simple dose‐volume constraint based optimization, the organ at risk's sensitivity is accounted for volumetrically and an estimated risk of toxicity can be assessed already during treatment planning. A gEUD cutoff for a certain organ at risk reduces biological effect to a single numeric value. Different DVHs, however, can lead to the same gEUD value. This can explain why the gEUD value in brainstem was met in some cases despite having NTCP >5%. Using gEUD during optimization can therefore be used as an estimate to guide sparing of OAR during the planning process. As a safe‐measure, subsequent NTCP calculations are recommended to evaluate effective NTCP for the individual treatment plan. This can be seen in Table 5. In our case, the radiation protocol deviates from the 2 Gy‐fractions, the original gEUD parameter sets were derived from. Hence, we recalculated the gEUD into a biologically equivalent gEUD, which can be considered as a close estimate of the true value.

Using models to estimate complication risks comes with pitfalls and limitations: the 3D patient dose is reduced to a 2D DVH, excluding spatial, anatomical, and physiological information. Next, the 2D graph is reduced to a single point of interest or to a model‐based NTCP.^35^ Hence, complex dosimetric and anatomic information is reduced to a single risk measure. Compared to models that rely only on single points in the DVH, however, the model used herein considers a large fraction of the DVH, which may be considered more radiobiologically logical. As one of the limitations, we had to base the computations on toxicity data available from tumor treatment in human radiation oncology. In veterinary radiation oncology, well‐curated toxicity data for intracranial organs at risk are not available. Hence, we herein assumed that similar organs such as brain and brainstem would react in a similar manner in dogs, taken into account the different relative volume parameters. We acknowledge that the organs at risk of different species might have different sensitivities toward radiation. As a second limitation, exact tumor type was not known but presumed from cross‐sectional imaging. Also, imaging characteristics of non‐neoplastic masses can sometimes mimic neoplasms. We therefore chose the margins for possible microscopic infiltration based on imaging criteria, rather than on tissue biopsies. We accounted for possible microscopic infiltration with a 2 mm CTV overlapping with brain and brainstem. Other reported GTV‐CTV margins range from 0 to 0.5 cm, pointing out a lack of knowledge and consensus for CTV margins in meningioma in dogs.^2^, ^4^, ^26^ Dogs have a higher prevalence of atypical or malignant meningiomas compared to humans (up to 43% versus 1.5%) with infiltrative growth pattern.^36^, ^37^ Histologically, microscopic infiltration into the normal brain and brainstem is described in 23‐43% of dogs, but exact distances of infiltration are unknown.^37^, ^38^, ^39^, ^40^ The present study included mainly presumed meningiomas. Tumors of glial origin are less common in the brainstem area of dogs and would most likely need larger GTV‐CTV margins to account for infiltration, according to human practice on gliomas.^37^, ^41^, ^42^ This would lead to larger PTV volumes, and again result in increased risk of toxicity when using a SIB protocol in glioma patients. Choice of appropriate margin is an inherent problem in radiation oncology, with a specific lack of consensus in veterinary radiation oncology.

In conclusion, the use of gEUD objectives as calculated for the present study provides a good concordance with the more laborious NTCP computations. In contrast to the latter, gEUD objectives are already available during the planning process and can influence optimization before finishing a radiation therapy plan. Radiation plans optimized with biologically based parameters can improve normal tissue sparing. With the higher dose from the proposed SIB IG‐IMRT protocol an acceptable risk of <10% was obtained in the majority of dogs according to our theoretical planning study, yielding a higher tumor control. This encourages and motivates us to use biologically based parameters for treatment planning in future clinical veterinary patients.

## LIST OF AUTHOR CONTRIBUTIONS

### Category 1

(a) Conception and Design: Meier, Besserer, Rohrer Bley
(b) Acquisition of Data: Meier, Rohrer Bley
(c) Analysis and Interpretation of Data: Meier, Besserer, Rohrer Bley

### Category 2

(a) Drafting the Article: Meier, Besserer, Rohrer Bley
(b) Revising for Intellectual Content: Meier, Besserer, Rohrer Bley

### Category 3

(a) Final Approval of the Completed Article: Meier, Besserer, Rohrer Bley

## CONFLICT OF INTEREST

The authors have declared no conflict of interest.
